# Prediction of clinical peanut allergy status among children in Hamilton, Ontario using chart review data collected during 2012–2015

**DOI:** 10.1186/s13223-017-0179-8

**Published:** 2017-02-08

**Authors:** Elizabeth Simms, Gary Foster, Katherine Arias, Mark Larché, Tosha Freitag, Tina Walker, Susanna Goncharova, Andrea Marrin, Andreas Freitag, Manel Jordana, Susan Waserman

**Affiliations:** 1 Michael G. DeGroote School of Medicine, St Joseph’s Hospital L314, McMaster University, 50 Charlton Avenue East, Hamilton, ON L8N 4A6 Canada; 2 Department of Clinical Epidemiology and Biostatistics, McMaster University, Hamilton, ON Canada; 3 AstraZeneca Canada, Mississauga, Canada; 4 Division of Allergy and Clinical Immunology, Department of Medicine, McMaster University, Hamilton, ON Canada; 5 McMaster Immunology Research Centre, McMaster University, Hamilton, ON Canada; 6 Department of Pediatrics, McMaster University, Hamilton, ON Canada; 7 Department of Medicine, McMaster University, Hamilton, ON Canada

**Keywords:** Peanut allergy, Skin prick test, Wheal size

## Abstract

**Background:**

Peanut sensitization does not necessarily indicate clinical peanut allergy, and uncertainty as to whether or not there is true peanut allergy can lead to increased anxiety and decreased quality of life for patients and their families. The gold standard for diagnosing clinical peanut allergy is the oral food challenge, but this method is time-consuming and can cause severe allergic reactions. It would therefore be beneficial to develop a tool for predicting clinical peanut allergy in peanut-sensitized individuals whose peanut allergy status is unknown so as to better determine who requires an oral food challenge for diagnosis.

**Methods:**

Two separate studies were conducted. In Study 1, we recruited 100 participants from the allergy clinic at McMaster University and community allergy outpatient clinics in the greater Hamilton area. We examined 18 different variables from participants and used univariate and multivariable logistic regression analysis to determine how well these variables, singly and in combination, were able to predict clinical peanut allergy status. In Study 2, we conducted a retrospective chart review of a second cohort of 194 participants to investigate the reproducibility of our findings. This was a matched case–control study where 97 peanut-allergic participants were gender- and age-matched to 97 non-allergic control participants.

**Results:**

Peanut skin prick test wheal size was the best predictor of clinical peanut allergy in both study cohorts. For every 1 mm increase in wheal size, the odds ratio of an individual having clinical peanut allergy was 2.36 in our first cohort and 4.85 in our second cohort. No other variable approached the predictive power of wheal size.

**Conclusions:**

Peanut skin prick test wheal size is a robust predictor of clinical peanut reactivity. The findings of this study may be useful in guiding clinician decision-making regarding peanut allergy diagnostics.

## Background

Peanut allergy is a serious public health concern, especially in westernized countries. Its prevalence has doubled in the past 10 years and currently stands at approximately 2% [1].

Peanut allergy is diagnosed by combining clinical history with diagnostic methods that may include skin-prick tests (SPT) and serum IgE measurements to peanut [2]. Many parents have avoided feeding their children peanut in an attempt to prevent peanut allergy, yet many children have developed sensitization to peanut, as demonstrated by a positive peanut SPT or peanut-specific IgE, and continue to avoid peanut. However, peanut sensitization does not necessarily mean clinical peanut allergy, and uncertainty as to whether or not there is true peanut allergy can lead to increased anxiety and decreased quality of life for patients and their families. The gold standard for diagnosing clinical peanut allergy is the oral food challenge, but this method is time consuming and requires proper set-up and personnel to manage potential severe allergic reactions [3]. Therefore, it would be beneficial to develop a tool for predicting clinical peanut allergy in peanut-sensitized individuals whose peanut allergy status is unknown so as to better determine who requires an oral food challenge for diagnosis.

The goal of this study was to use clinical and laboratory data from patients of known peanut allergy status to develop a statistical model to predict clinical peanut allergy in peanut-sensitized individuals. To determine its predictive merit, the model was applied to a group of patients with positive skin prick tests, but unknown clinical reactivity because they had never knowingly ingested peanut. These patients of unknown clinical status then underwent an oral peanut challenge to determine their true peanut allergy status and this outcome was compared to their model-predicted peanut allergy status.

## Methods

### Study 1: patient recruitment and data collection

100 participants were recruited from the allergy clinic at McMaster University and community allergy outpatient clinics in the greater Hamilton area.

All participants were at least 6 years of age and of either sex. Exclusion criteria for the study were uncontrolled or severe asthma, receipt of allergy injections in the past, and use of injectable epinephrine 1 month prior to the start of the study. Individuals taking daily antihistamines, leukotriene receptor antagonists, or nasal, inhaled, or oral corticosteroids were also excluded. These interventions may have interfered with our study measurements, particularly cytokine secretion.

We collected the following data on each participant: age, sex, peanut SPT wheal size, clinical peanut allergy status, peanut ImmunoCAP, total IgE, supernatants from peripheral blood mononuclear cells (PBMC) under unstimulated and peanut-stimulated conditions, immediate family history of peanut allergy, asthma, rhinitis, and eczema status.

Participants were divided into 4 groups according to their peanut allergy status based on history and peanut skin prick test.

Group 1 consisted of peanut allergic individuals. These individuals had a prior history of an allergic reaction to peanut on ingestion and a positive SPT to peanut. Allergic symptoms included, but were not limited to, urticaria, angioedema, dyspnea, cough, wheeze, nausea, vomiting, lightheadedness, rash, and/or shock.

Group 2 consisted of individuals who had a positive skin prick test to peanut, but could tolerate peanut ingestion without difficulty. Thus, these individuals were not allergic to peanut and their skin test results were designated as “false positives”.

Group 3 consisted of individuals who had a positive skin prick test, but no known history of peanut ingestion. Many of these individuals may have avoided peanut for specific reasons, such as a family history of peanut allergy. It was therefore uncertain whether they would react to peanut on ingestion and they were considered to be at risk of clinical reactivity based on the presence of sensitization.

Group 4 consisted of individuals who had a negative skin prick test to peanut and had previously ingested peanut without problems. Consequently, they served as a negative control group. This group did not have any other food or environmental allergies.

This study was approved by the Research Ethics Board at McMaster University and all participants, or their guardians, provided written informed consent.

#### Skin prick test measurements

The forearm was prepped with alcohol and peanut extract (ALK-Pharmaceuticals, Mississauga, ON, Canada) was applied to the skin of the dorsal forearm. A sterile metal lancet (HollisterStier, Spokane, WA, USA) was used to puncture the skin below the allergen droplet. Skin prick test wheal size was measured after 15 min. Tape was placed on the dorsal forearm and an outline of the wheal was traced. The widest diameter of the wheal was measured by two different study nurses.

#### Peanut and total IgE plasma measurements

Total IgE was measured using the Immage 800 (Beckman Coulter, Mississauga, ON, Canada) and peanut-specific IgE antibodies were measured using the Phadia 250 (Thermo Scientific, Waltham, MA, USA).

#### Cytokine measurements

Mononuclear cells were isolated from 30 to 40 ml of blood by density gradient centrifugation after red blood cells were lysed with AKC lysis buffer. Cells were re-suspended in RPMI supplemented with 10% FBS, 1% l-glutamine, 1% penicillin/streptomycin, 55 µM 2-mercaptoethanol (Thermo Scientific), 1 mM sodium pyruvate, 10 mM HEPES and 0.1 mM MEM NEAA (Thermo Scientific). Viable cells were counted via Trypan Blue (Thermo Scientific) exclusion and re-suspended at 8 × 10^6^ cells/mL. 125,000 live cells per well were cultured in triplicates in medium alone or with 50 µg/mL/well of crude peanut extract in flat-bottom 96-well plates (BD Biosciences, Mississauga, ON, Canada). After 5 days of culture at 37 °C and 5% CO_2_, the triplicates were pooled, spun down and cell-free supernatants harvested and stored at −80 °C until further analysis. Cytokines in cell-free supernatants were quantified using Luminex (Millipore Canada Ltd, Etobicoke, ON, Canada) following the manufacture’s instructions.

### Statistical analysis

Each predictor was entered into a univariate logistic regression analysis to determine if it was associated with the primary outcome—clinical peanut allergy status. We then generated cumulative models composed of multiple predictors using multivariable logistic regression. All univariate and multivariable analyses included the 69 study participants from Groups 1, 2, and 4.

For all models, parameter estimates were obtained for each predictor and expressed as odds ratios with corresponding 95% confidence intervals and associated *p* values. *p* values are reported to 4 decimal places.

Hierarchical models were compared to determine if the model with the greater number of predictors was statistically significantly better at predicting the primary outcome than the model with fewer predictors. This was done by comparing the models’ −2 Log Likelihood statistics. For each model, the area under the Receiver Operating Characteristics (ROC) curve was reported as a measure of discriminability. The best model was used to predict the peanut allergy status of participants in Group 3 and to determine the predicted probability (Pr) of each participant having clinical peanut allergy. Using Pr, we classified each individual as having a peanut allergy or not based on a specific cutpoint. We chose this cutpoint to eliminate false negatives and maximize true positives in the data set.

All analyses were conducted in SAS version 9.4.

#### Peanut challenges

All individuals in Group 3 underwent a peanut challenge to determine peanut allergy status. The food challenge took place in the Allergy Clinic at McMaster University Medical Centre under the supervision of a study physician. A research/Critical Care nurse and study physician were present at all times with the appropriate set-up to deal with any and all allergic reactions.

All subjects had baseline vital signs taken, body weight measured, and an intravenous inserted prior to oral food challenge.

Each subject was given either 1 mg of peanut or placebo orally mixed with grape jelly or applesauce. Peanut flakes were the source of peanut and cracker crumbs were used as the placebo. The dose of peanut was increased to 5 mg and increased every 15–30 min to 10, 25, 50, 100, 250, 500 mg, 1, and 2.5 g until the maximum dose of 2.5 g was reached or objective findings of allergic reaction were observed. 2.5 g is the equivalent of 5 peanuts.

Subjects were carefully observed for the following signs of allergic reaction: rash (erythema, morbilliform rash, urticaria, angioedema), ocular (conjunctival swelling, scleral edema, tearing), nasal (congestion, rhinorrhea, sneezing), respiratory (wheezing, cough, drop of PEF or FEV1 by >20%), gastrointestinal (vomiting, diarrhea, abdominal pain), systemic (blood pressure drop by >20%).

Vital signs (oxygen saturation, blood pressure, heart rate, respiratory rate) were assessed before each dose, with every new symptom reported, and when objective findings were observed.

If a subject developed any two mild symptoms (generalized itchiness or flushing, runny nose, watery eyes, or sneezing) or any one severe symptom (persistent cough, significant abdominal pain, nausea, vomiting, diarrhea, swelling of the lips or face, difficulty breathing, wheezing, or fainting) the challenge was immediately stopped and the subject was considered to be peanut allergic [4].

Subjects who experienced allergic reactions were treated with appropriate medications, namely intramuscular epinephrine, intravenous antihistamines, and corticosteroids (1 mg/kg for 3 days). The subjects were observed for 4–8 h after an allergic reaction to ensure that it had been adequately treated and resolved [5].

If 2.5 g of peanut was tolerated, 10 g was administered in an open challenge and subjects were monitored for signs of allergic reaction. In the event of a reaction, each subject received appropriate medication and monitoring.

The results of the oral peanut challenges were then compared to patients’ predicted peanut allergy status.

### Study 2: patient recruitment and data collection

We conducted a retrospective chart review of a separate cohort of 194 subjects: 97 with confirmed clinical peanut allergy, and 97 sex- and age-matched controls without clinical peanut allergy. Peanut allergy was defined as: the participant had consumed peanuts in the past and displayed peanut allergy-compatible symptoms, as described earlier, and had undergone confirmatory testing. For each participant, we collected date of birth, sex, peanut skin prick test wheal size, allergic rhinitis, asthma, and eczema status. We also recorded food allergy status for milk, egg, wheat, individual nut, and nut mix.

### Statistical analysis

The predictive value of each variable was analyzed using exact conditional logistic regression. All analyses were conducted in SAS version 9.4.

## Results

### Study 1: participant characteristics

A total of 100 subjects participated in this study and a summary of their characteristics is displayed in Tables 1, 2. Half of the participants were female, 14% had an immediate family member (parent or sibling) with a peanut allergy, and 59% had a comorbid allergic condition (asthma, rhinitis, or eczema). The median age in years was 15.5, but there was an imbalance in age between Groups 1, 2, and 4 versus Group 3. In Group 3 the median age was 9, whereas the median age of Groups 1, 2, and 4 was 21.

**Table 1:** Study groups

Variable	Group 1 (n = 30)	Group 2 (n = 17)	Group 3 (n = 31)	Group 4 (n = 22)
Clinical peanut allergy status	Y	N	U	N
Positive skin prick test to peanut	Y	Y	Y	N

Y = confirmed clinical peanut allergy; N = confirmed not allergic to peanut; U = unknown

**Table 2:** Clinical characteristics

Variable	Groups 1 (n = 30)	Group 2 (n = 17)	Group 3 (n = 31)	Group 4 (n = 22)
Age (y)^a^	14.17 (6.20)	25.82 (16.00)	10.16 (3.12)	36.23 (10.82)
Wheal size (mm)^a^	7.50 (3.35)	3.97 (1.15)	5.48 (1.91	0 (0)
Sex (F)	14 (47)	11 (65)	10 (32)	15 (68)
Father with allergy	0 (0)	0 (0)	1 (3.2)	0 (0)
Mother with allergy	2 (2.9)	0 (0)	1 (3.2)	0 (0)
Sibling with allergy	2 (7)	1 (6)	8 (26)	0 (0)
Asthma	18 (60)	5 (29)	17 (55)	0 (0)
Rhinitis	11 (37)	12 (71)	8 (26)	0 (0)
Eczema	9 (30)	0 (0)	13 (42)	0 (0)

All other entries are n (%)

^a^Entries are mean (standard deviation)

#### Predictive values of individual variables

For the 69 participants in Groups 1, 2, and 4, each of 18 predictor variables was entered as a single predictor of the primary outcome—clinical peanut allergy status. Nine variables were selected for entry into the multivariable model based on a univariate *p* value <0.1 (Table 3). All other variables evaluated had a *p* value >0.1. Using area under the ROC curve (AUC) as a measure of discriminability between peanut allergic and non-peanut allergic participants, peanut SPT wheal size was the best univariate predictor, with AUC = 0.927. In other words, peanut SPT wheal size was the variable most able to accurately predict true clinical peanut allergy. A larger wheal size was associated with a stronger risk of clinical peanut allergy: the odds ratio for each 1 mm increase in wheal size was 2.36.

**Table 3:** Univariate logistic regression analysis of peanut allergy predictors

Variable	OR [95% CI]	*p* value	AUC
Wheal size	2.362 [1.533, 3.639]	<0.0001	0.927
Peanut IgE	1.083 [1.021, 1.149]	0.0080	0.812
Total IgE	1.001 [0.999, 1.002]	0.0749	0.822
Gender (M)	2.286 [0.859, 6.082]	0.0978	0.600
Asthma (Y)	10.199 [3.105, 33.511]	0.0001	0.736
IL-13 (P)	1.002 [1.000, 1.003]	0.0138	0.729
IL-5 (M)	1.000 [0.999, 1.002]	0.4921	0.539
IL-5 (P)	1.002 [1.001, 1.004]	0.0040	0.696
IL-9 (P)	1.0012 [0.9999, 1.003]	0.0621	0.650

n = 69

#### Generation of predictive models

**Fig. 1:**
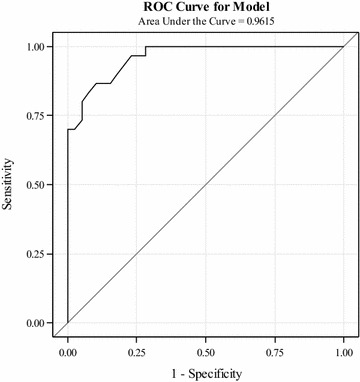
Model 1 receiver operating curve

The results of the univariate analyses revealed that peanut skin prick test wheal size was the strongest single predictor of peanut allergy status among all variables assessed. The area under the ROC curve for wheal size was significantly better than any other single predictor. Next, we entered wheal size into the model and added each of the remaining variables one at a time in a stepwise process. We found that rhinitis significantly improved the model by increasing the area under the ROC curve and improving the model’s ability to predict true peanut allergy. Following the same strategy, we found that asthma was the only remaining predictor that was statistically significant when added to the model. We called this combination of peanut wheal size, rhinitis, and asthma “Model 1”. No other predictors were significant (*p* < 0.1) when added to Model 1. In Model 1, wheal size and asthma were positively related to peanut allergy status, but rhinitis was curiously protective. The AUC for model 1 was 0.962, demonstrating an improvement in prediction over any single variable analyzed above. Table 4 shows the results of this model. The ROC curve for Model 1 is shown in Fig. 1.

**Table 4:** Multivariable logistic regression results for model 1

Variable	OR [95% CI]	*p* value
Wheal size	2.606 [1.517, 4.477]	0.0005
Rhinitis (Y)	0.084 [0.010, 0.688]	0.0209
Asthma (Y)	6.278 [0.962 40.975]	0.0549

n = 69

#### Using model 1 to predict clinical peanut allergy status in group 3

Applying Model 1 to Group 3 generated a probability of peanut allergy (Pr) for each of the 31 participants with a positive skin prick test but unknown clinical peanut allergy status. We chose a cutpoint value for Pr that would minimize false negatives and maximize true positives, thereby maximizing the model’s ability to predict true peanut allergy. Using a cutpoint of 0.35 for Pr, we classified participants into two groups: if Pr ≥ 0.35, the individual was predicted to have a peanut allergy, and if Pr < 0.35, the individual was predicted to not have a peanut allergy. When predicted peanut allergy status was cross tabulated with known peanut allergy status based on peanut challenge, Model 1 predicted Group 3 peanut allergy status well, but made four errors. It predicted a negative result (NOT allergic to peanuts) for two participants whose oral food challenge indicated they did have clinical peanut allergy. One of these subjects had a wheal size equal to 5 mm, and the other had a wheal size equal to 6 mm. It also predicted a positive result for two participants who did not have an oral food challenge reaction.

We then removed asthma from the model in order to examine whether sensitivity would improve. In our study, we defined sensitivity as the proportion of patients with a known peanut allergy who the model correctly predicts as having a peanut allergy. We called this new model comprised of only wheal size and rhinitis “Model 2” and used it to predict Group 3 peanut allergy status. Model 2 proved to have a sensitivity of 100%, as it correctly predicted every participant with a known peanut allergy as having clinical peanut allergy. However, only four of seven participants with known negative peanut allergy status were correctly predicted as having no clinical allergy, indicating a specificity of 57.1% (Table 5). We defined specificity as the proportion of patients with a known negative peanut allergy status who the model correctly predicts as not having a peanut allergy. Three participants with known negative peanut allergy status were incorrectly predicted to have a peanut allergy. We believe this is an acceptable type of error, as it does not carry the same risk as classifying an allergic subject as non-allergic. The wheal sizes of these three participants were 3, 4 and 5 mm.

**Table 5:** Sensitivity and specificity of Model 2

Predicted allergy status	Known allergy status		
	Positive [n (column %)]	Negative [n (column %)]	Total [n (column %)]
Positive	24 (100)	3 (42.9)	27 (87.1)
Negative	0 (0)	4 (57.1)	4 (12.9)
Total	24	7	31

When applied to Group 3, Model 2 (using a cutpoint of 0.35) correctly predicted the clinical peanut allergy status of 24/24 allergic individuals, indicating a sensitivity of 100%.

### Study 2: evaluating the predictive value of wheal size and allergic rhinitis status in a second patient cohort

We conducted a retrospective chart review of 97 participants with confirmed clinical peanut allergy and 97 sex- and age-matched control participants without clinical peanut allergy. Their clinical characteristics are summarized in Tables 6, 7.

**Table 6:** Participant demographics

Variable	Peanut allergic	Non-peanut allergic
Age in years [mean (SD); min, max]	9.87 (4.46); 3.00, 20.90	9.86 (4.45); 3.10, 20.80
Wheal size in mm [mean (SD); min, max]	8.62 (4.00); 2.00, 20.00	0.28 (0.97); 0.00, 5.00
Female sex [n (%)]	47 (48.5)	47 (48.5)

**Table 7:** Clinical characteristics

Variable	Peanut allergic	Non-peanut allergic
Allergic rhinitis status	57 (58.8)	54 (55.7)
Non-allergic rhinitis status	4 (4.1)	7 (7.2)
Asthma status	43 (44.3)	22 (22.7)
Eczema status	27 (27.84)	26 (26.80)
Egg sensitization	9 (9.28)	7 (7.22)
Milk sensitization	2 (2.06)	10 (10.31)
Wheat sensitization	1 (1.03)	0 (0)
Nut sensitization	33 (34.02)	13 (13.40)
Nut mix sensitization	19 (19.59)	7 (7.22)

All entries are n(%) for affirmative

We classified allergic and non-allergic subjects according to wheal size (Table 8).

**Table 8:** Peanut skin prick test wheal size by peanut allergy status

Wheal size (mm)	Clinical peanut allergy status		
	No	Yes	Total
0	89	0	89
1	0	0	0
2	1	1	2
3	4	4	8
4	2	7	9
5	1	8	9
≥6	0	77	77
Total	97	97	194

We analyzed the predictive value of each variable using exact conditional logistic regression. This analysis revealed a linear dependency among variables when wheal size was entered into the model. Because of this, we were unable to obtain a parameter estimate for wheal size.

We then analyzed the predictive value for each of the predictor variables using exact simple logistic regression (Table 9). We found wheal size to be the best predictor of clinical peanut allergy, with an odds ratio of 4.85 for every 1 mm increase in wheal size. The area under the ROC curve was 0.995, with a *p* value of <0.0001. The predictive value of wheal size was so dominant that no other variable was statistically significant when added to a model that included wheal size.

**Table 9:** Exact simple logistic regression univariate analysis

Variable	Odds ratio [95% CI]	*p* value	−2 Log L	Area under ROC
Wheal size	4.85 [2.859, 11.44]	<0.0001	33.306	0.995
Rhinitis	1.13 [0.618, 2.086]	0.7717	268.752	0.515
Non-allergic rhinitis	0.55 [0.115, 2.270]	0.5368	268.063	0.515
Eczema	1.05 [0.533, 2.082]	1.0000	268.915	0.505
Egg allergy	1.31 [0.415, 4.345]	0.7950	268.668	0.510
Milk allergy	0.18 [0.019, 0.901]	0.0329	262.767	0.541
Nut allergy	3.31 [1.548, 7.444]	0.0012	257.237	0.603
Nut mix allergy	3.11 [1.176, 9.248]	0.0191	262.329	0.562

We then examined the sensitivity and specificity of wheal size at different cutpoints, ranging from 1 mm to 5 mm (Table 10). Sensitivity reached 100% at 1 mm, while specificity reached 100% at 5 mm.

**Table 10:** Sensitivity and specificity for wheal size cut-offs

Wheal size (mm)	Sensitivity (%)	Specificity (%)
>1	100	91.8
>2	99.0	92.8
>3	94.9	96.9
>4	87.6	99.0
>5	79.4	100

## Discussion

In Study 1 we analyzed the ability of eighteen different variables, alone and in combination, to predict clinical peanut allergy in peanut-sensitized individuals. Our results show that peanut SPT wheal size is by far the best predictor of peanut allergy. While the univariate analysis identified peanut-specific IgE, total IgE, male sex, asthma, and IL-5, IL-9 and IL-13 responses to peanut as being significant predictors of clinical reactivity, subsequent multivariable analyses found these variables to be related to peanut SPT wheal size and thus when entered into a model with wheal size were non-significant.

The analysis of our initial patient cohort revealed peanut SPT wheal size as the best univariate predictor, with an AUC of 0.927. For every 1 mm increase in wheal size, the odds ratio of an individual having a clinical peanut allergy was 2.36.

In this cohort, wheal size was positively associated with peanut allergy status while the presence of rhinitis was curiously protective. Using Model 2, that included both wheal size and rhinitis, we were able to successfully predict the clinical peanut allergy status of 100% of allergic subjects. However, the model misclassified three non-allergic subjects as allergic.

Interestingly, rhinitis was found to be protective against clinical peanut allergy in our first group of subjects. However, we were unable to reproduce this finding in our second patient cohort. To our knowledge, there are no other studies in the literature reporting a similar protective effect of allergic rhinitis in peanut allergy. This unexpected finding may have been an anomalous result caused by a small patient cohort size.

The strong predictive value of wheal size emerged in the analysis of our much larger second patient cohort. We found that for every 1 mm increase in wheal size, the odds ratio of an individual having clinical peanut allergy was 4.85. No other variable approached the predictive power of wheal size.

Other models of clinical peanut allergy prediction, such as The Cork Southampton Predictive Index [6], have used peanut SPT, serum specific IgE, total IgE, sex, and age to predict clinical peanut allergy. However, our data sets did not identify any variables that could reproducibly improve on the predictive ability of SPT wheal size in our patient cohorts.

The strong association between SPT wheal size and clinical peanut allergy has been described elsewhere. The HealthNuts longitudinal food allergy study in Melbourne, Australia, reported that a wheal size of 8 mm had 95% positive predictive value for clinical peanut allergy in 1-year-old infants [7]. Decreasing wheal size was associated with peanut allergy resolution in these patients at age 4, while increasing wheal size was associated with persistence [8]. Other groups have reported wheal size cut-points from 4 to 15 mm reaching 100% specificity when used to predict clinical peanut allergy [9, 10].

The skin prick test does produce false positive results that can lead to misclassification of non-allergic patients as allergic. False positive results emerged in our study, and have been reported previously [9, 11, 12]. Food allergy misdiagnosis negatively impacts the quality of life of patients and their families to the same degree as true peanut allergy. Heightened anxiety associated with eating, disruption of daily activities, and the need to carry an epinephrine auto-injector is common to both groups [13]. However, it is our strong belief that it is never acceptable to misclassify an allergic patient as non-allergic, especially in the context of a potentially severe diagnosis such as peanut allergy. A sensitive screening test, such as the SPT, is preferable to one that sacrifices sensitivity in favour of specificity.

One potential weakness of this study is the restricted range of variables it examined. Specifically, we did not include component testing or the basophil activation test, both of which are emerging tools in the field of food allergy research [14–19]. At the time of the study these tests were not available to us. Additionally, they are not without limitations and are not currently the standard of care in peanut diagnostics.

Component resolved IgE testing for Ara h 1, 2, and 3 has been highlighted as more predictive of clinical allergy than whole peanut-specific IgE, and sensitization to Ara h 2 emphasized as particularly discriminatory. However, there is a lack of consensus on appropriate component testing cutoffs and sensitivity and specificity measures of different cutpoints vary widely between studies. Reported sensitivity for a commonly used cutpoint of 0.35 kU/L ranges from 60 to 100% and specificity ranges from 72 to 96%. Beyer et al. [15] described a 90% probability for positive peanut challenge at 14.4 kU/L, and a cutpoint of 42.2 kU/L was required to reach a 95% probability. Additionally, the importance of individual components varies regionally, testing lacks standardization across commercial kits, and individual patient outcomes can deviate from component-associated correlations [20].

The basophil activation test has been proposed as a useful diagnostic tool for peanut allergy, but its broad utility is limited by its requirement for fresh blood and variability in basophil activity between individuals [21]. As with component testing, the cost of the basophil activation test limits its use in routine clinical practice.

The comprehensive statistical analyses used in this study consistently highlighted the superior ability of peanut SPT wheal size to predict clinical reactivity to peanut. SPT wheal size emerged as the dominant predictor of peanut allergy in both univariate and multivariable analyses in two separate patient cohorts. Our statistical analyses also determined that the predictive power of IgE laboratory measurements, both total and peanut-specific, were not independent of wheal size. This was also true for peanut-induced Th2 cytokine production from peripheral blood mononuclear cells. When added to any statistical models containing wheal size, the predictive power of all laboratory measurements became statistically non-significant. As such, the power of peanut SPT wheal size to predict clinical peanut allergy was dominant and reproducible.

## Conclusions

Peanut skin prick test wheal size is a robust predictor of clinical peanut reactivity. We have found that patients with a wheal size of <1 mm do not display clinical peanut allergy and patients with wheal sizes ≥6 mm are clinically reactive. In patients with wheal sizes between 1 and 5 mm inclusive, oral food challenge may be appropriate to determine allergic status if history is indeterminate. Further studies of a large cohort of patients with wheal sizes in this range may be warranted. The findings of this study may be useful in guiding clinician decision-making regarding peanut allergy diagnostics.

## Abbreviations

SPT: skin prick test
PBMC: peripheral blood mononuclear cells
